# One-Step Fabrication of Microchannels with Integrated Three Dimensional Features by Hot Intrusion Embossing

**DOI:** 10.3390/s16122023

**Published:** 2016-11-29

**Authors:** Mike Debono, Dan Voicu, Mohammad Pousti, Muhammad Safdar, Robert Young, Eugenia Kumacheva, Jesse Greener

**Affiliations:** 1 Department of Chemistry, University of Toronto, Toronto, ON M5S 3H6, Canada; mdebono92@hotmail.com (M.D.); dan.voicu@utoronto.ca (D.V.); ekumache@chem.utoronto.ca (E.K.); 2 FlowJEM Inc., Toronto, ON M5S 3H6, Canada; info@flowjem.com; 3 Département de Chimie, Université Laval, Québec, QC G1V 0A6, Canada; mohammad.pousti.1@ulaval.ca (M.P.); muhammad.safdar@uef.fi (M.S.); 4 Department of Chemistry, University of Eastern Finland, Joensuu FI-80101, Finland

**Keywords:** microfluidics, microfabrication, thermoplastic, hot embossing, intrusion embossing

## Abstract

We build on the concept of hot intrusion embossing to develop a one-step fabrication method for thermoplastic microfluidic channels containing integrated three-dimensional features. This was accomplished with simple, rapid-to-fabricate imprint templates containing microcavities that locally control the intrusion of heated thermoplastic based on their cross-sectional geometries. The use of circular, rectangular and triangular cavity geometries was demonstrated for the purposes of forming posts, multi-focal length microlense arrays, walls, steps, tapered features and three-dimensional serpentine microchannels. Process variables, such as temperature and pressure, controlled feature dimensions without affecting the overall microchannel geometry. The approach was demonstrated for polycarbonate, cycloolefin copolymer and polystyrene, but in principle is applicable to any thermoplastic. The approach is a step forward towards rapid fabrication of complex, robust, microfluidic platforms with integrated multi-functional elements.

## 1. Introduction

Thermoplastic-based microfluidic devices have a range of application-selective material properties and bonding methodologies [1,2,3]. Their physical robustness enables reliable fluidic and probe interfacing, embedded valves, as well as stable operating conditions, even under high pressures [3,4,5]. Cost still remains the bottleneck for higher penetration of these devices into the growing global market, especially for designs that require integration of functional and/or complex features. New microfabrication techniques that overcome this barrier will open a range of applications that benefit from integrated three-dimensional features. These include devices with tunable surface hydrophobicity [6]; optimized surface reactions [7]; ability to support surface acoustic waves [8]; resistance to biofouling, controlled cell growth, non-specific adsorption [9,10,11,12], and integrated optical elements [13,14]. Spot heating using laser pulses have been shown to generate pillars due to expansion of molten thermoplastic with some control of their size based on laser intensity, but suffers from large variances in feature morphology [15]. Grey-scale photolithography has been demonstrated in the creation of three-dimensional (3D) features, but is not widely utilized because of the need for specialized photomasks [13,14,16,17,18]. Material printing [18,19,20,21,22] and computer numerical controlled (CNC) micro machining [23] are becoming versatile options for rapid prototyping of 3D microfluidics. However, in the case of CNC machining, surface finish is typically rough. A recent explosion in 3D printing technologies is making impressive impact in consumer product fabrication markets. The microfluidic community is closely monitoring the advancements, [18,21] but for now, with the exception of two-photon polymerization methods, 3D printing feature resolution lateral directions is still roughly two orders of magnitude less than photolithography. In addition, both CNC machining and printing rely on a sequential approach to the formation of features, with resolution decreasing with fabrication speeds, making them more time-consuming and unfavourable for mass fabrication of microfluidic parts compared to templating methods, for the time being. In hot embossing and injection moulding, thermoplastics heated near to or above their glass transition temperatures can rapidly conform to template features with excellent fidelity. In these approaches, the hurdle becomes the fabrication of the template, which must be redone for each iteration during the prototyping process [24,25,26,27]. A variation on hot embossing called hot intrusion embossing (or partial embossing) offers a solution to this problem. Typically, with the use of a single 2D imprint template and proper calibration of embossing conditions, penetration of heated thermoplastic into cavities is varied, thus achieving control over feature height. However, after its demonstration as a means to produce templates for elastomeric microfluidic devices, there has been no significant development for this promising approach to be used for the direct fabrication of thermoplastic microfluidic devices [28].

Here we significantly expand on the concept of hot intrusion embossing by demonstrating a one-step method to fabricate thermoplastic microchannels containing integrated controllable microfeatures. We demonstrate that imprint templates containing microcavities with specific cross-section geometries locally control penetration velocity of heated polymer. The approach led to channels with integrated features such as posts, microlens arrays, semi-occluding walls, staircase patterns and features with 3D tapered edges. To demonstrate its versatility, the approach was implemented in different thermoplastics. As a proof-of-principle, we fabricated microchannels during one intrusion embossing step, containing two separate embedded functional regions: a 3D serpentine channel for mixing and a microlens array, featuring lenses with different focal lengths.

## 2. Experimental

### 2.1. Materials

Imprint templates were produced by FlowJEM Inc. (Toronto, ON, Canada). Thermoplastics used in this study included polycarbonate (PC, Lexan 9034-112, Sabic Polymershapes, Mississauga, ON Canada); cycloolefin polymer (COP, 1420, Zeon Chemicals L.P., Louisville, KY, USA); and polystyrene (PS, UVF Non-glare, Plaskolite Inc., Columbus, OH, USA). Glass transition temperatures were 145 °C, 138 °C, 100 °C, respectively.

### 2.2. Template Fabrication 

Details regarding the experimental setup, materials used and characterization techniques are given in the Supplementary Materials. Imprint templates were fabricated based on a photolithographic approach using masks that contained opaque and transparent regions, corresponding to recessed and raised features, in the final imprint templates, respectively [29]. Each imprint template consisted of multiple raised, elongated features for microchannels with one inlet and one outlet. Within each raised channel structures on the imprint template, were a series of smaller cavities with different cross-sectional geometries: circular, rectangular and triangular. The height of the microchannel template feature was the same as the cavity feature depth, which was equal to the thickness of the photoresist layer in the imprint template.

### 2.3. Fabrication Parameters

Hot intrusion embossing (referred to simply as “embossing”) was implemented inside a custom vacuum chamber located within a temperature controlled hydraulic press (Model 3851-C Carver Inc., Wabash, IN, USA). Temperature control was ±1 °C accuracy for top and bottom platens and included liquid leads for rapid cooling. The target thermoplastic was put in contact with the imprint template within the chamber which was then evacuated and the temperature was then raised to the embossing temperature (*T*_e_). Following a 5 min temperature stabilization period, embossing pressure (*P*_e_) was applied for *t*_e_ = 2 min. Cooling for approximately 30 s was applied until de-embossing temperature (*T*_d_) was reached. Finally, the vacuum was broken and de-embossing was achieved with the aid of a blade to apply light prying action between the thermoplastic substrate and the imprint template. Afterwards, the thermoplastic substrate retained the exact inverse features of the imprint template everywhere, except in the microcavity region where the height was determined by the degree of polymer intrusion. Due to the reduced interaction between the intruding polymer and the microcavities, de-embossing was much easier than for fully embossed samples. In principle, we speculate that this should have the effect of extending the life-time of the photoresist imprint template. Further details of the embossing process are given in the Supplementary Materials.

### 2.4. Imaging

Three-dimensional profiler measurements were acquired using an optical profilometer (NT1100, Veeco, Oyseter Bay, AZ, USA). Two-dimensional images of the embossed thermoplastic devices were acquired using an optical microscope (BX41, Olympus, Melville, NY, USA), with a CCD camera capture system (Evolution–VF, MediaCybernetics, Silver Spring, MD, USA) and image analysis of captured micrographs was conducted by software (Image-Pro Plus 5.0, Media Cybernetics, Silver Spring, MD, USA and ImageJ open-source program). Images of microfeatures were also acquired using a grazing angle camera system (32°) attached to a drop shape analyser (DSA 100, KRÜSS GmbH, Hamburg, Germany). Three-dimensional characterization of microlenses was done by confocal laser scanning microscopy (FV1200, Olympus, Richmond Hill, ON, Canada), using a 10× objective and by atomic force microscopy (Nanoscope III Multimode, Digital Instruments, Santa Barbara, CA, USA).

### 2.5. Numerical Simulations

Estimations of polymer melt flow velocity through the microcavities in the embossing templates were achieved by 3D finite element modeling (COMSOL Multiphysics, COMSOL Inc., Burlington, MA, USA). The simulation results were normalized so that relative differences in flow velocity within a single cavity could be made based on estimations of *P*_e_, *T*_e_ and viscosity. Velocity profiles along the feature lengths were extracted for each model cavity shape. Microlens focal lengths were estimated by computer simulation software (COMSOL Multiphysics). This was accomplished by analyzing lens dimensions as measured by confocal laser scanning microscopy and atomic force microscopy. In order to reduce the computational cost, a 2D simulation was conducted on lens cross-sections. The refractive index for all lenses was chosen as *n* = 1.58 and *n* = 1.33 for a water filled channel. All optical simulations were operated at an incident light wavelength of 500 nm. 

## 3. Results

### 3.1. Hot Intrusion Using Cylindrical Microcavities

**Figure 1:**
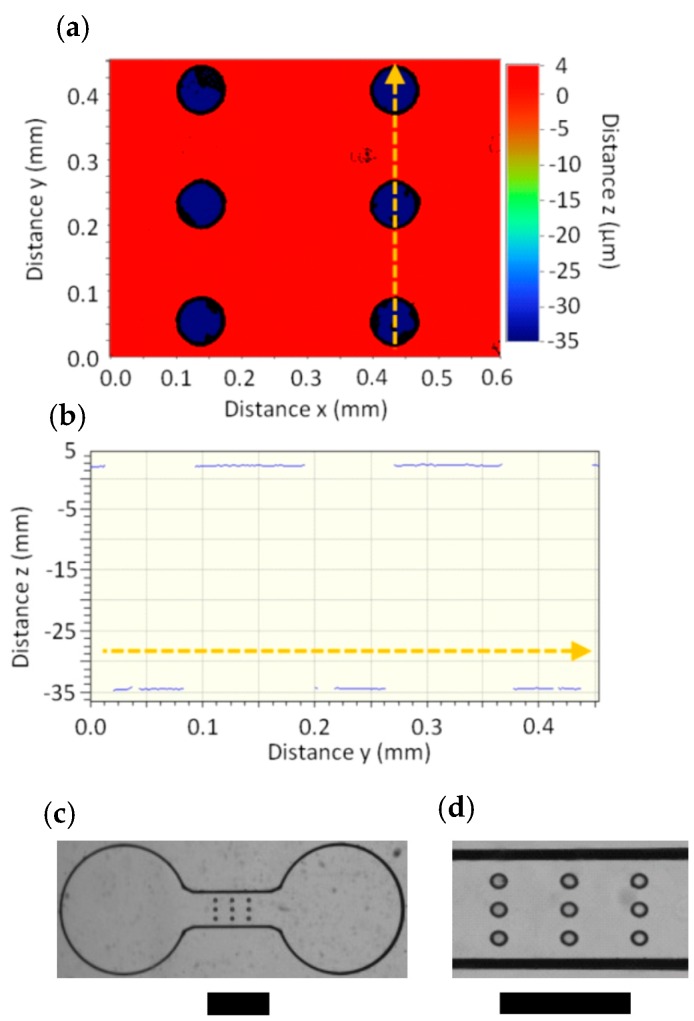
(**a**) Three-dimensional profiler measurements of IT_1_ in the vicinity of the array of cylindrical cavities (*d*_h_ = 80 μm). Channel width was 640 µm. The bottom of the cylindrical cavities are blue, 38.5 ± 0.2 µm below the top surface of the imprint template. (**b**) Two-dimensional height profile along the cross-section in (**a**). The direction indicated by the arrows in (**a**) and (**b**) are the same. Optical micrograph of the embossed PC device (*T*_e_ = 175 °C, *P*_e_ = 1.7 MPa, *t* = 2 min) moulded from IT_1_ with imaging through a 1.25× objective (**c**) and a 4× objective (**d**). Scale bars in (**c**) and (**d**) are 1 mm and 500 µm, respectively.

In the first experiment, the imprint template (IT_1_) contained a 3 × 3 array of cylindrical microcavities in the main microchannel feature, which were 38.5 ± 0.2 µm deep. The cavities had hydraulic diameter *d*_h_ = 80 µm, which for cylindrical cavities is equal to its cross-section diameter. Profiler measurements for a subsection of this array are shown in Figure 1a,b. After embossing, the resulting cylindrical micropillars appeared as small circles from the top view (Figure 1c,d). A second imprint template (IT_2_) also had cylindrical microcavities but was 44 ± 0.2 μm deep. See Supplementary Materials for more details.

For a particular embossing time, the height of the pillars was determined by the polymer melt flow velocity (*ν*_f_) into the cavities, whereas the overall channel depth was defined solely by the height of the channel features on the imprint template and not on embossing conditions. When the viscous flow dominates the capillary flow (see Supplementary Materials for calculation), the filling velocity (*ν*_f_) should increase with *d*_h_ and *P*_e_ and decreases with *η_T_* [27,30]. In some cases the depth of the cavity could play a role due to trapped air, but experiments here were done under vacuum to avoid such complications [31]. In this proof of concept work, we rely on empirical relationships between process parameters and velocity of the heated polymer, because the system can only control processing variables (temperature, pressure) outside the home-built embossing chamber, which likely differs from those applied to the substrate and template (Supplementary Materials). For many polymers, including those used in this work, semi-empirical equations accurately predict the sharp decrease of *η_T_* with increasing *T*_e_ [32,33].

**Figure 2:**
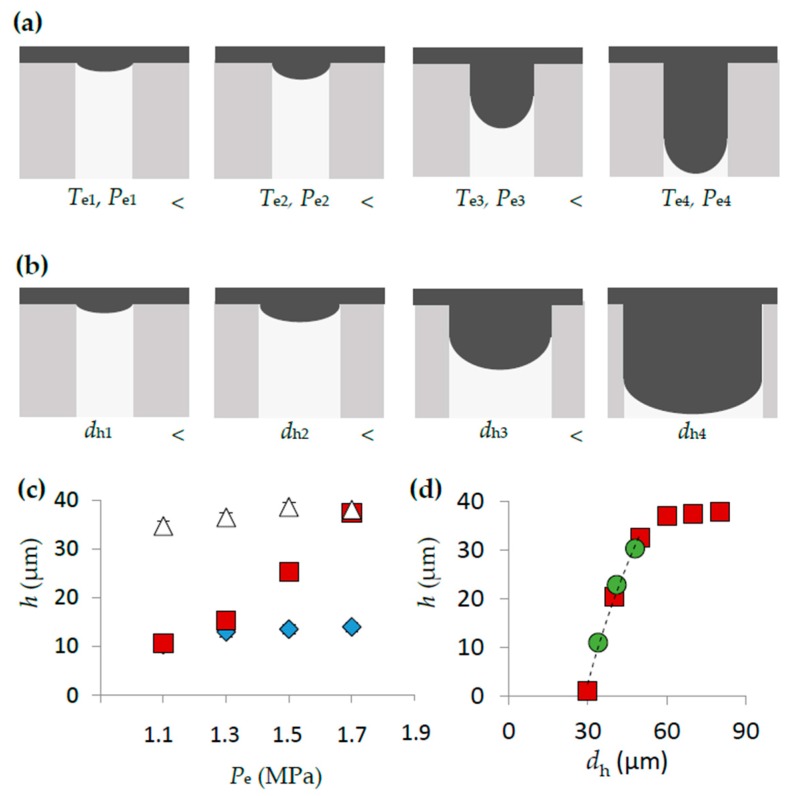
(**a**) Cartoons depicting the progressive filling of one cylindrical cavity in the imprint template with the polymer melt (grey) as *T*_e_ is increased from *T*_e1_ to *T*_e4_ and/or *P*_e_ is increased from *P*_e1_ to *P*_e4_. (**b**) Changes to the filling of a cavity with changes to hydraulic diameter from *d*_h1_ to *d*_h4_ for a particular *T*_e_, *P*_e_ set. (**c**) Variation in *h* posts versus *P*_e_ in the range 1.1 MPa to 1.7 MPa for *T*_e_ = 165 °C (triangles), *T*_e_ = 170 °C (squares), *T*_e_ = 175 °C (diamonds) for devices fabricated in PC. (**d**) Plot of normalized *h* vs. *d*_h_ for pillars fabricated using the imprint template IT_1_ (red squares) and IT_2_ (green circles) with embossing conditions were *T*_e_ = 115 °C and *P*_e_ = 1.5 MPa in PS. The dashed trend line is the result of fitting the data to a second-order polynomial for points where *h*_c_ − *h* ˃ 1.5 µm, with *R*^2^ = 0.991. Data points were generated from the average from nine separate measurements. Error bars were calculated as the standard deviation of nine separate measurements, but were smaller than the data points.

Figure 2a,b shows two different ways to achieve controlled intrusion of the heated polymer into cavities. The first is by controlling embossing conditions *T*_e_ or *P*_e_. An increase to either results in increases to *ν_f_* within the cylindrical cavities (and corresponding increases to *h* of the final embossed features). The second approach to control intrusion is to change the *d*_h_ of the microcavities (Figure 2b). The first method was used as a calibration step to determine an appropriate combination of *T*_e_ and *P*_e_ for control over the polymer intrusion (Figure 2c). We note that if the *T*_e_ is too high, full intrusion was achieved into the cavity for all applied *P*_e_ (traditional embossing), whereas if it was too low, no value of *P*_e_ could result in appreciable intrusion. In the second approach where cavity diameters in the range 30 μm ≤ *d*_h_ ≤ 100 μm were used controlled *h*. We demonstrated the generalizability with two imprint templates with different *h_c_* values (*h*_c1_ = 38.5 μm, *h*_c2_ = 44 μm). The mask and embossed features from the two imprint templates are shown in the Supplementary Materials. The relationship between *d*_h_ and *h* in PS (*T*_e_ = 115 °C and *P*_e_ = 1.5 MPa) generated from both imprint templates, hown in Figure 2d, is represented by the empirical relation:

(1) $$h=-0.0427d_{h}^{2}+0.496d_{h}-108.99$$

We note that despite the differences in *h_c_* of the two templates, the *h* versus *h_c_* trend is nearly the same for most values of *h*. For *h_c_* − *h* < 2 µm *h* becomes nearly constant with *d*_h_, due to the interaction of the heated polymer with the far side of the cavity. Equation (1) enabled prediction of pillar heights as a function of cavity diameter. This work demonstrates the potential for a generalizable approach that gives quantitative design rules that can predict the height of any structure. For this to be realized, a full multivariable fitting process relating *h* to all control variables (*T*_e_, *P*_e_, *d*_h_, *t*_e_) for each target thermoplastic material is required. 

### 3.2. Hot Intrusion in Non-Cylindrical Cavities

**Figure 3:**
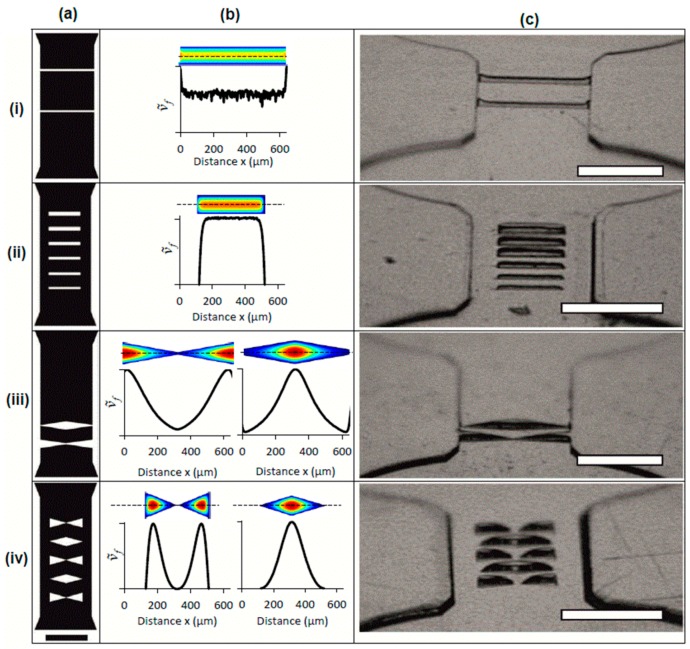
Two-dimensional template design (**a**) and corresponding *ṽ_f_* simulation in one and two dimensions (**b**), and embossing results (**c**). Close up of imprint template schematics at the feature region for (i) partially occluding straight walls with widths 20 µm (bottom) and 25 µm (top); (ii) free-standing staircase features with widths (bottom to top) 25 µm, 30 µm, 35 µm, 40 µm, 45 µm and 50 µm; (iii) tapered occluding walls and (iv) free-standing tapered features. Results from numerical simulations of *ṽ_f_* in microcavities formed with masks in (**a**) are shown adjacent to the relevant design (i–iv) in (**b**). Colour scale ranges from *ṽ*_f_ = 0 (dark blue) to *ṽ*_f_ = 1 (red) are for qualitative comparison only. Velocity profiles were acquired along the length of the microcavity cross-section at its middle (marked with a dashed line). Grazing angle images of PS microchannels (**c**) that correspond to imprint template schematics in (**a**). Embossing conditions to produce images in (**c**) were: *P*_e_ = 1.5 MPa and *T_e_* = 110 °C (i), *P*_e_ = 1.5 MPa and *T*_e_ = 105 °C (ii), *P*_e_ = 1.5 MPa and *T*_e_ = 105 °C (iii) and *P*_e_ = 1.5 MPa and *T*_e_ = 110 °C (iv). Common scale bar for (**a**) is 500 µm. All scale bars for images in (**c**) are 500 µm and all channels were 640 µm wide.

Next, we demonstrated the use of imprint templates containing rectangular and triangular cavities to produce complex 3D microstructures in microchannels. Cavity shape and size resulted in different local *d*_h_, permitting control over *ν*_f_ within a single microcavity feature. Figure 3 is comprised of three subfigures which include the two-dimensional designs of the imprint templates with different microcavity geometries (Figure 3a), a two-dimensional simulation of the normalized intrusion velocity, *ṽ*_f_, within a microcavity feature and a plot of *ṽ*_f_ along an indicted cross-section (Figure 3b), and grazing angle images of the microfabricated parts in PS using a stamp (Figure 3c) bearing the same geometries as in Figure 3a. The latter demonstrates how selectively attenuated polymer flow through specific micro cavity geometries can be used to fabricate in-channel features such as partially occluding straight walls, free-standing staircase features, tapered occluding walls and free-standing tapered features. We note that the velocity maps are intended to qualitatively describe the range of polymer penetration velocities through a single microcavity based on the local geometry. This cannot necessarily be linked to the structure of the final embossed feature. For example in the case that the polymer flow front encounters the far side of the cavity, it will form a flattened shape and redistribution of polymer flow will ensue. This can be seen in embossing results for designs ii–iv, for example. Due to its relevance to biological sciences, we used PS for the remainder of the work.

### 3.3. Walls and Stairs Using Rectangular Microcavities

Long, narrow trenches that cut perpendicularly across the raised microchannel feature in the imprint templates (*h*_c_ = 44 μm) resulted in partial polymer intrusion over long distances. Figure 3a(i) shows the schematic of an imprint template with two wall feature cavity widths (*w_i_* = 20 and *w_ii_* = 25 µm) intersecting the microchannel. Simulations predicted nearly constant *ν*_f_ along the length of the trench, except near their intersection with the microchannel side-walls, where larger local *d*_h_ resulted in higher local *ν_f_* (Figure 3b(i)). Embossing PS with *T*_e_ = 110 °C and *P*_e_ = 1.5 MPa resulted in wall heights of 28.7 µm and 34.0 µm for the feature cavities with *w_i_* and *w_ii_*, respectively (Supplementary Materials). Increased wall heights were observed at the intersection with the microchannel side wall as predicted by simulation. As seen in Supplementary Materials, reducing the temperature to *T*_e_ = 105 °C resulted in decreased wall heights (12.5 µm and 16.4 µm, respectively) due to increases to *η_T_*.

Next, rectangular features with different *w* were demonstrated for the fabrication of a staircase pattern. Figure 3a(ii),b(ii) show the schematic of a microfluidic device with six rectangular cavities located within a microchannel and the simulated local *ṽ*_f_ within one of the microcavities, respectively. Each rectangular cavity was 400 μm long, with widths in the range of 25 μm ≤ *w* ≤ 50 μm at 5 μm intervals. The results from embossing PS (*T*_e_ = 105 °C and *P*_e_ = 1.5 MPa) show a series of free-standing rectangular features with heights between 8 μm and 44 μm (Figure 3c(ii)). Lower *ν_f_* near the edges of the features were predicted by simulation due to reduction in the local *d*_h_. 

### 3.4. Tapered Features Using Triangular Microcavities

Next we explored the use of triangular microcavities to impose continuous gradients in the local *d_h_* along the feature length, which could systematically vary local *v*_f_ and feature *h*. Figure 3a(iii) shows a design with two elongated triangular features (*θ*_apex_ = 15°) that intersected the channel cross-section. Two variations were arranged, either with their most narrow part of the triangular microcavity (*w* = 13 μm) or the widest part (*w* = 100 μm) meeting in the middle of the microchannel. The gradual changes to *ṽ_f_* along the length of the feature (Figure 3b(iii)) resulted in features with tapered edges in the embossed PS sheet, using *P_e_* = 1.5 MPa and *T_e_* = 105 °C (Figure 3c(iii)). The embossing was repeated for *T*_e_ = 110 °C and *T*_e_ = 115 °C, which produced features with larger portions of the cavity filled by thermoplastic, leaving an increasingly constructed orifice feature at the most narrowed regions. This opens the way for producing 3D orifice structures with tuned dimensions.

Finally, we used disconnected triangular cavities (Figure 3a(iv)) to create free-standing structures with tapered edges (Figure 3c(iv)). The features ranged in width from *w* = 100 μm to *w* = 0 μm (*θ*_apex_ = 34°). The *d*_h_ near triangle apexes resulted in zero local penetration, leading to strongly tapered wall edges. Cavities with tip to tip separation resulted in two separate PS features.

### 3.5. One-Step Fabrication of Integrated Element 1: Three-Dimensional Serpentine Channels

**Figure 4:**
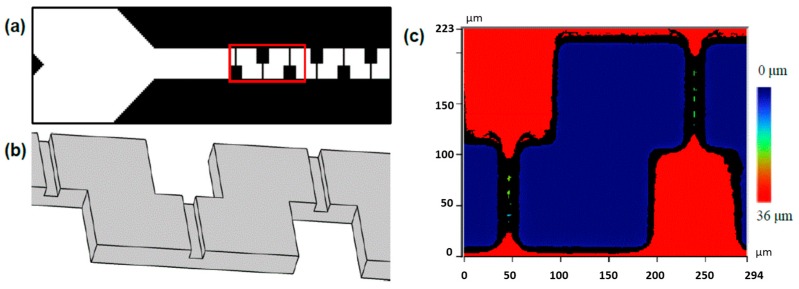
(**a**) Portion of the design file used to create the mask for a three-dimensional mixer. Y-channel intersection at the left side of the image feeds into downstream mixing compartment. (**b**) A three-dimensional rendering of the expected results from intrusion embossing for the portion of the channel highlighted in red in (**a**). The channel interior volume (grey) shows 3D switchback in the x, y plane with channel constrictions in the z-direction. (**c**) Optical profilometry from embossed PS at *T*_e_ = 110 °C, *P*_e_ = 1.4 MPa. The un-embossed surface (red) with depressions that formed the microfluidic channel (blue) and intra-channel constrictions in the z-direction (green).

A microfluidic device with two functional elements was fabricated using the current method. The first element was a three-dimensional serpentine channel sequence that could be used for mixing. Fabrication of three-dimensional serpentine channels are typically far more complicated than for two-dimensional channels. This is because the former can require double sided wafer etching, aligned two layer photolithography and/or three-dimensional writing techniques [34,35,36,37]. Here, we demonstrated a design that uses localized intrusion embossing as a one-step fabrication method for three-dimensional switchback channels. Figure 4 shows the the design used to generate the appropriate imprint template and a three-dimensional rendering of the target channel volume. Embossing in PS was undertaken at *T*_e_ = 110 °C, *P*_e_ = 1.4 MPa with optical profiler results shown in Figure 4c. The approach also has the potential to form other passive mixing structures, such as chaotic mixers [38].

### 3.6. One-Step Fabrication of Integrated Element 2: Multi-Focal Length Microlenses

**Figure 5:**
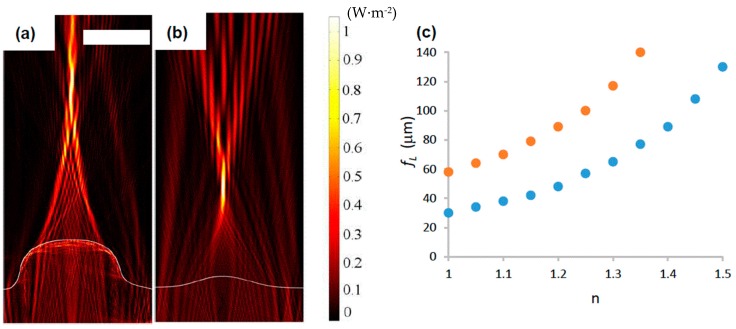
The trace of two x, z lens profiles (white lines) acquired by CLSM (**a**) and AFM (**b**), produced by cylindrical cavities with *d*_h_ = 37.5 µm and *d*_h_ = 30 µm, respectively. Embossing conditions were *T*_e_ = 110 °C, *P*_e_ = 1.4 MPa and *t* = 2 min in PS. Indices of refraction were *n*_PS_ = 1.58 and *n*_air_ = 1. Simulation results superimposed of the normalized electromagnetic power density, (W·m^−2^), resulting from a 500 nm light source placed at the bottom of the lens. Scale bar is 20 µm. The dependency of focal length on n is shown in (**c**) for lenses formed in cavities with *d*_h_ = 37.5 µm (orange) and *d*_h_ = 30 µm (blue).

In a downstream segment of the same microfluidic device shown in the preceding section, hot intrusion embossing into an array of cylindrical cavities produced microlens arrays. The lens focal lengths (*f_L_*) were varied based on the *d*_h_ of the cavities. In Figure 5a,b, two measured x, z profiles of lenses with *d*_h_ = 37.5 µm, *d*_h_ = 30 µm are shown. Using indices of refraction *n*_PS_ = 1.58 and *n*_air_ = 1.33 and the measured dimensions of the lens, a finite element simulation calculated an electromagnetic energy density map, giving *f_L_*. Figure 5c shows the effect of channel filling with liquids with different values of n on *f_L_*, for the two lenses in Figure 5a,b. See Supplementary Materials for corresponding optical images.

## 4. Conclusions

This work addresses the need for a cost-effective, one-step method to fabricate thermoplastic micro-channels containing complex micro features. We used a rapidly fabricated imprint template by photolithography with cavities that attenuated the intrusion of heated polymer during embossing based on their cross-sectional geometries. Fabrication was conducted in polycarbonate, cycloolefin copolymer and polystyrene, but in principle, can be extended to any thermoplastic. This approach enabled control over the in-channel micro feature height and geometry, while the channel height itself remained constant in all cases. The approach significantly advances the use of hot intrusion embossing by offering design rules for the fabrication of complex 3D structures. A numerical simulation provides a qualitative understanding of the filling velocities into different parts of the imprint template microcavities due to the local geometries. With a careful selection of microcavity geometries, fabrication of posts, multi-focal length microlense arrays, walls, steps, tapered features and three-dimensional serpentine microchannels was demonstrated. This work opens the way for applications requiring mixing, sorting, textured surfaces, cell manipulation and embedded optical sensors. In addition, applications requiring three-dimensional channel constrictions could be benefited, including burst valves for centrifugal microfluidics, blood constriction models. The technique can benefit from empirical studies and rigorous simulations to fine-tune cavity geometries and operating conditions to expand the range of applications [39]. Finally, we note that the approach is compatible with low-cost photoresist-based stamps.

## Supplementary Materials

The Supplementary Materials containing materials, methods and simulations is available online at http://www.mdpi.com/1424-8220/16/12/2023/s1.
