# Supplementary material for: One-Step Fabrication of Microchannels with Integrated Three Dimensional Features by Hot Intrusion Embossing
Source: Sensors (Basel). 2016 Nov 29;16(12):2023. doi: 10.3390/s16122023 (PMC5191004; doi:10.3390/s16122023)
Supplement: Supplementary file 1 [file sensors-16-02023-s001.pdf]

Supplementary Materials: One-Step Fabrication of Microchannels with Integrated Three Dimensional Features by Hot Intrusion Embossing

Mike Debono, Dan Voicu, Mohammad Pousti, Muhammad Safdar, Robert Young, Eugenia Kumacheva and Jesse Greener

Hot Embossing

The imprint template was placed on top of a 1 mm thick silicone rubber sheet at the bottom of the vacuum chamber, with its features facing up. A thermoplastic substrate was placed on the top of the embossing features of the imprint template. A rigid sheet of copper was placed on top of the thermoplastic sheet and a second silicone rubber sheet was placed on top of that. The rubber sheets served as a precautionary measure, redistributing any pressure gradients that may arise during embossing. As a result, however, thermal losses were expected to occur through the silicone rubber sheets, the thermoplastic and the imprint template. Thus we expected the set temperature of the platens to be higher than the actual embossing temperature (the temperature at the interface between the imprint template and thermoplastic sheet). In addition, the compression of the silicone rubber sheets and an o-ring component located in the vacuum chamber assembly absorb some pressure from the applied pressure of the hydraulic press. This resulted in an embossing pressure being lower than that which is reported by the gauge on the hydraulic press. Every embossing cycle consisted of heating the platens to embossing temperature, 5 min of heating of the imprint template and thermoplastic substrate within a vacuum chamber with no applied pressure, application of embossing pressure for 2 min at embossing temperature, followed by rapid cooling to the de-embossing temperature.

Imprint Templates with Cylindrical Microcavities

In this study, we used two imprint templates with cylindrical microcavities. The first (IT_1) was used to create 3 × 3 arrays of round pillars with diameters of 80, 70, 60, 50, 40, and 30 µm, as shown in the main paper, describing Figures 1–4. Cavity height for IT_1 was h_c = 38.5 µm ± 0.2 µm. A second imprint template (IT_2) contained cavities of cylindrical pillars and other shapes to demonstrate the versatility in fabricating micro features with different heights in the same channel. Cavity heights for IT_2 were h_c = 44 µm ± 0.2 µm.

Capillary Number

In general, flow through a capillary is either viscosity-driven or capillary-driven. Here we justify the claim in the main paper that viscous flow dominates capillary flow. In capillary-driven flow, the interfacial tension is a key value in determining the flow velocity, whereas it does not play an appreciable role in viscosity-driven flow. The capillary number (Ca) is a measure of the relative effect of viscous forces to capillary forces and is given in Equation (1):

$$Ca = \eta_T v/\sigma \tag{1}$$

where η_T is the viscosity, v is the polymer melt flow velocity, and σ is the surface tension. A high Ca value means that the flow is dominated by viscous forces. Using the values $\eta_T = 1.5 \times 10^7$ (Pa·s), $v = 0.5$ (µm·s^{-1}), and $\sigma = 0.033$ (N·m^{-1}), we see that Ca >> 1, and the flow of the polymer melt through the cylindrical micro cavities in the imprint template is determined to be dominated by viscous flow. Therefore the relationship of polymer flow into the cylindrical cavities in the imprint template given by Equation (1) (main text) is independent of interfacial tension between the polymer melt and the imprint template surface.

Control and Calibration of Hot Intrusion Embossing Using T_e and P_e

Next, for the purposes of optimization of the technique, we examined the effect of T_e and P_e on the heights of micro pillars in different thermoplastic polymers. In each case, the devices were fabricated using the same imprint template with cylindrical cavities of d = 80 μm. Figure S1 shows the resulting heights of the pillars formed in (a) polycarbonate (PC) and (b) cycloolefin polymer (COP) at different temperatures and pressures. For PC and COP, we identified a temperature and a pressure range that enabled control of h, over the range 10 μm ≤ h ≤ 38.5 μm. For PC, the choice of embossing temperature of 170 °C enables control over pillar height by changing pressures in the range 1.1 MPa ≤ P_e ≤ 1.7 MPa (Figure S1a). Similarly, we used T_e = 170 °C for COP to control the pillar height by changing pressures in the range 0.75 MPa ≤ P_e ≤ 1.1 MP (Figure 3b). For polystyrene (PS) we determined that in the range of 110 °C ≤ T_e ≤ 115 °C control over pillar heights 2.5 μm ≤ h ≤ 38.5 μm could be altered by applying pressures 0.55 MPa ≤ P_e ≤ 1.7 MPa (data not shown). Outside these temperature ranges, control of pillar height was not possible for the values of P_e attempted in the ranges listed above for each material. We note that control over pillar heights could also be achieved by maintaining constant embossing pressure and varying the temperature. For example, Figure S1a shows that for P_e = 1.5 MPa, the variation of the temperature in the range 165 °C ≤ T_e ≤ 175 °C yielded pillars with heights from 13 to 37 μm. (We verified that channel height remained 38.5 μm for each experiment).

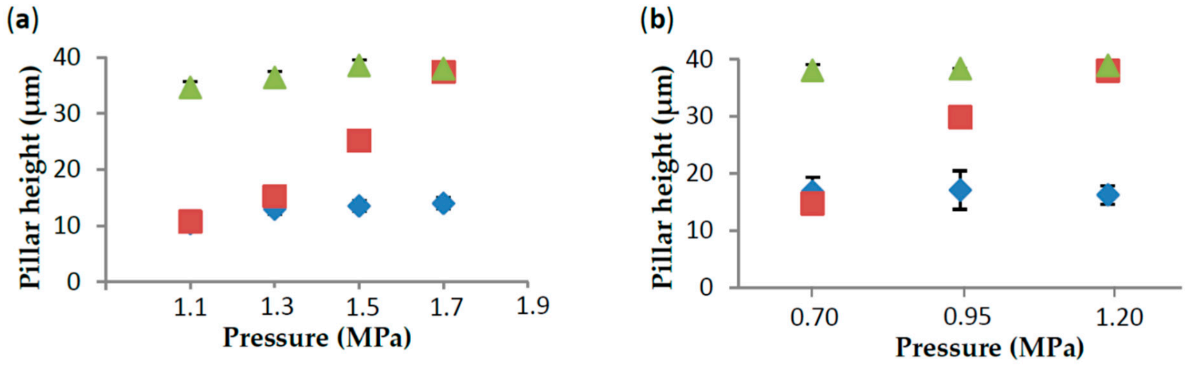

Figure S1. (**a**) Variation in h versus P_e in the range 1.1 MPa to 1.7 MPa for T_e = 165 °C (triangles), T_e = 170 °C (squares), T_e = 175 °C (diamonds) for devices fabricated in PC. (**b**) Variation in h with P_e in the range 0.75 MPa to 1.1 MPa for T_e = 155 °C (triangles), T_e = 170 °C (squares), T_e = 185 °C (diamonds) for devices fabricated in COP. In each case the full height of the MF channel was 38.5 μm. Each data point was the result of averaging nine separate measurements, whereas error bars were generated from their standard deviation. Some error bars are smaller than the data points.

Control of Hot Intrusion Embossing Using *d*

A range of cylindrical cavity diameters used to generate micropillars with controllable heights were generated using 2 different imprint templates (IT_1 and IT_2, having heights being 38.5 μm and 44 μm, respectively). Figure S2a,c shows the two designs from each IT along with an example of the embossed result (b), (d) and (e). We showed the results of the normalized pillar heights in Figure 2d (main paper). We note that the largest d_h, which resulted in the deepest cavity intrusion of heated polymer had tops which were flattened due to contact with the far side of the capillary.

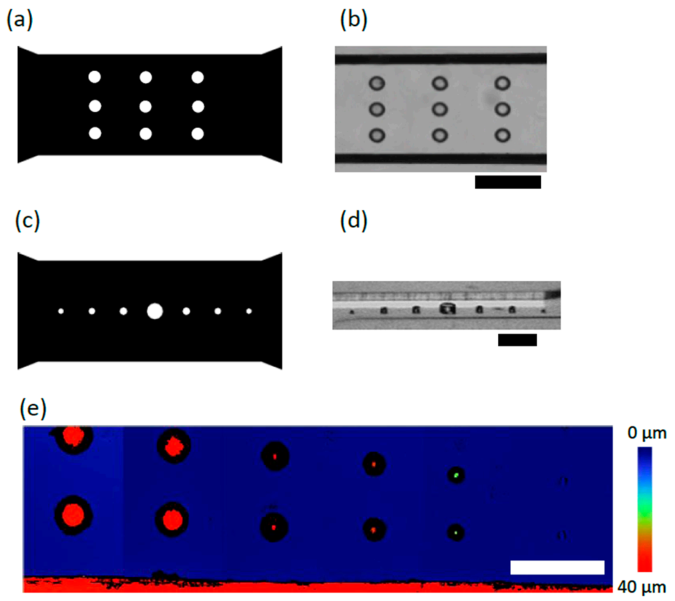

Figure S2. (**a**) Schematic of imprint template 1 (IT_1) with d = 80 µm with black and white representing raised and recessed features, respectively (circular inlet and outlet are not shown). Each IT_1 contained separate channels with four different 3 × 3 arrays each, for d equal to 30, 40, 50, 60, 70, 80 µm. (**b**) Image of embossed device (PS) in the same region. Schematic of IT_2, featuring seven symmetrically arranged cylindrical cavities with d equal to 34, 41, 48, 100, 48, 41, 34 µm. (**d**) A microscope image taken at grazing angle, showing the imprinted PS channel segment and pillars corresponding to (**c**). For both (**a**) and (**c**) hot embossing of PS was conducted at T_e = 115 °C and P_e = 1.5 MPa. (**e**) Composite 3D colour map of a part of the hot embossed PS microfluidic channel using IT_1 in the region of the micro pillars from six different regions of IT_1 with pillar diameters that ranged from 80 µm (i) to 30 µm (vi). Scale bar is 200 µm.

Characterization of Physical Dimensions of Microlenses

Using low pressure and temperature, microlenses were produced by strongly limiting thermoplastic intrusion into the microcavities. We characterized microlens dimensions sing a combination of atomic force microscopy (AFM), optical profilometry and confocal laser scanning microscopy (CLSM). AFM yielded high resolution, continuous vertical cross-section profiles, but data acquisition was very slow and was limited to a 6 µm maximum vertical tip deflection (Figure S3a). Optical profilometry, could give fast data acquisition over a relatively large surface area, but suffered from missing data points from the sides of the microlenses, which reflected light away from the instrument's objective lens (Figure S4b). CLSM on the other hand produced continuous, three-dimensional data sets with relative ease (e.g., Figure 5a in the main paper). Data files were transformed from three-dimensional image stacks to vertical cross-section profiles using the "reslice" and "find edge" commands in ImageJ and then saving as XY coordinates using the "wand" tool. Therefore, CLSM and AFM results were used to generate vertical cross-sections which were subsequently used for simulation of focal lengths.

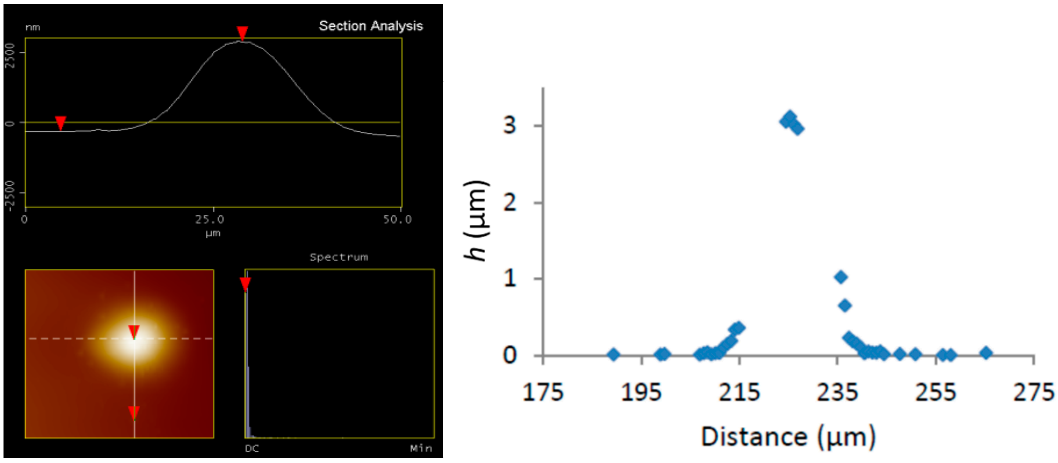

(**a**) (**b**)

Figure S3. (**a**) Output from AFM software showing vertical cross-section profile and 3D representation of a lens in polystyrene created in a d = 30 µm cavity, with T_e = 101 °C and P_e = 700 lbs. (**b**) Profile along one axis of a similar microlens as (**a**) with an optical profiler.

Simulation of Microlens Focal Length

For building the 2D geometry of lenses, the interpolated curve of the coordinates extracted from CLSM and AFM measurements with relative tolerance less than 0.01 is used to avoid convergence problems during simulation. Refractive index of n = 1.58 was used for all lenses. All simulation were operated at an incident light wavelength of 500 nm and a free triangular mesh with maximum element size of 100 nm were used. A beam was introduced to the system from the bottom of the lens and a scattering boundary condition is used on the wall to avoid back reflectance of light. For analyzing the result, the normalized power flow, time average of the beam was plotted versus the Cartesian coordinates which for a two dimensional system can be acquired from the Equation (2):

$$P = \sqrt{P_x^2 + P_y^2} \quad (2)$$

where P_x and P_y are x and y components of power flow, time average respectively.

Temperature Dependence on Height of Partially-Occluding Straight Walls

As discussed in the main paper, thin trenches could be used to place partially blocking walls in microchannels in order to locally modify the channel depth. Figure S4 shows the results from a mask designed to produce a channel with thin trenches of 20 and 25 µm. As discussed in the main paper, the small difference in width resulted in different wall heights, depending on the embossing conditions. The heights of the walls were measured to be 28.7 (i) µm and 34.0 µm (ii). Using the same imprint template and embossing at the same pressure (P_e = 1.5 MPa), a second PS microfluidic device was fabricated with T_e = 105 °C. The higher viscosity resulted in slower flow into the microtrenches leading to wall heights of 12.5 µm (i) and 16.4 µm (ii).

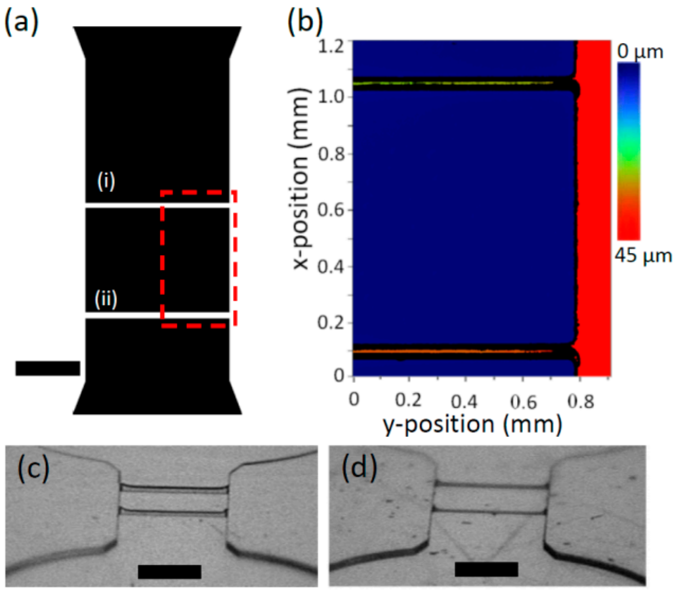

Figure S4. (**a**) Schematic of the segment of the imprint template with two trenches intersecting the microchannel (circular inlet and outlets are not shown). Black and white represent raised and recessed features on the imprint template, respectively. Channel height was the same as the micro trench depth, h_c = 44 µm. The width of the walls (i) and (ii) was 20 µm and 25 µm, respectively. Scale bar is 1 mm. (**b**) Optical profiler measurement of the PS microchannel from embossing using T_e = 110 °C and P_e = 1.5 MPa. Optical images at grazing angle showing the walls formed using P_e = 1.5 MPa and T_e = 110 °C (**c**) and 105 °C (**d**), respectively. Scale bars in (**c**) and (**d**) are 1 mm.

Lens Array

Figure S4 shows the array of cylindrical microcavities used to produce microlens arrays with different focal lengths. Colours blue and red showing the positions that were measured for Figure 5 in the main text.

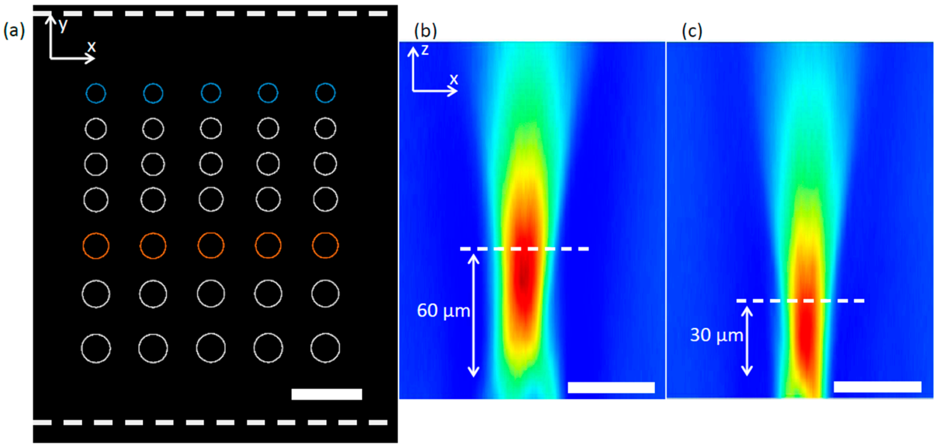

Figure S5. (**a**) Portion of the microchannel containing cylindrical cavities used for intrusion embossing of microlens array with multi focal lengths. Colours match Figure 5b. Dashed lines show microchannel walls. Scale bar is 100 µm. (**b**,**c**) Resliced vertical stacks showing light intensity in the *x*, *z* plane using a regular transmission microscope via imaging with changes to the focal planes by 5 µm steps. Vertical distance of 60 and 30 µm are superimposed for reference to Figure 5c. False colour was added using the "Physics" look up table in ImageJ. Scale bars in (**b**,**c**) are 50 µm.
